# Mobile Mind Mapping and Training Interventions for Non-communicable Disease Prevention in Primary Healthcare: A Systematic Review

**DOI:** 10.7759/cureus.107771

**Published:** 2026-04-26

**Authors:** Chinmaya Choudhury, Manjubala Dash, Jayestri Kurushev, M. Navaneetha

**Affiliations:** 1 Department of Nursing, Mother Theresa Post Graduate and Research Institute of Health Sciences, Pondicherry University, Puducherry, IND; 2 Department of Obstetrics and Gynaecology, Mother Theresa Post Graduate and Research Institute of Health Sciences, Pondicherry University, Puducherry, IND; 3 Department of Mental Health Nursing, Mother Theresa Post Graduate and Research Institute of Health Sciences, Pondicherry University, Puducherry, IND; 4 Department of Community Health Nursing, Pondicherry Institute of Medical Sciences, Pondicherry University, Puducherry, IND

**Keywords:** community health workers, mind mapping, mobile learning, non-communicable diseases, primary healthcare

## Abstract

Background: Non-communicable diseases (NCDs) impose a substantial burden on health systems, particularly in low-and middle-income countries where primary healthcare resources are limited. Community health workers and community health officers contribute to early detection, risk reduction, and health promotion; however, conventional training approaches may not adequately support knowledge retention and practical competencies. Evidence on integrating mind mapping with mobile-based learning remains limited.

Methods: This systematic review, conducted in accordance with Preferred Reporting Items for Systematic Reviews and Meta-Analyses (PRISMA) guidelines, evaluated training interventions for community-based health personnel in NCD prevention. Literature published between 2013 and 2025 was identified through PubMed, Scopus, and Google Scholar. Studies were screened using predefined criteria, and findings were synthesised using qualitative thematic analysis.

Results: Ten studies were included. Community-based interventions improved screening coverage, risk-factor awareness, and follow-up care. Educational interventions enhanced knowledge, communication competence, and self-efficacy. Innovative approaches, including mind mapping and simulation, improved cognitive organisation and understanding of complex information. Variability in study design and outcomes limited direct comparison.

Conclusions: Training interventions improve competencies in NCD prevention. Integration of mobile learning with visual strategies such as mind mapping shows potential to enhance training effectiveness and workforce capacity. Further high-quality studies are required to evaluate combined approaches and their impact on healthcare outcomes.

## Introduction and background

Non-communicable diseases (NCDs) are a leading cause of global morbidity and mortality, placing a substantial burden on healthcare systems, particularly in low- and middle-income countries where resources are often limited [1,2]. The increasing prevalence of conditions such as cardiovascular diseases, diabetes, cancers, and chronic respiratory diseases continues to challenge healthcare systems in terms of prevention, early detection, and long-term management [2,3]. Primary healthcare plays a critical role in addressing these challenges by serving as the first point of contact for individuals and enabling early identification, preventive interventions, and continuity of care [4-6].

Community health workers and community health officers are integral to strengthening primary healthcare systems, particularly in rural and underserved settings [7,8]. They contribute to NCD prevention and control through activities such as community outreach, household screening, lifestyle counselling, and follow-up care, which help bridge gaps between communities and formal healthcare services [9-11]. Their close interaction with communities allows early identification of at-risk individuals and supports improved adherence to treatment and lifestyle modifications [10-12]. Despite their critical role, gaps persist in the effectiveness of existing training approaches, which are often limited in their ability to enhance knowledge retention, critical thinking, and practical application required for managing complex health conditions [13-15].

Traditional training methods often rely on lecture-based approaches that may restrict learner engagement and limit the development of analytical and problem-solving skills [16,17]. Emerging educational strategies, such as mind mapping (a visual learning technique that organises information into structured diagrams showing relationships between concepts) and mobile-based learning (the use of digital platforms to deliver flexible, on-demand training through mobile devices), have shown potential in improving learning outcomes and accessibility of training. Mind mapping facilitates the organization and visualization of complex information, enhancing comprehension and retention [18,19], while mobile learning enables flexible and continuous access to educational content, particularly in resource-constrained settings [19,20].

Despite these advancements, existing literature predominantly examines community health worker training, mind mapping, and mobile learning as separate domains, with limited evidence on their integration within training programs for community-based health personnel in NCD prevention. For example, while community health workers may receive training on screening and counselling, and separate studies demonstrate the benefits of mind mapping or mobile learning, few studies evaluate how these approaches can be combined within a single training framework. This gap highlights the need for a focused synthesis of evidence to better understand how these approaches can be combined to strengthen training and improve workforce capacity in primary healthcare settings. Accordingly, this review aims to synthesize existing evidence on community health worker-led interventions and educational strategies, and to examine the potential integration of mind mapping and mobile-based learning for enhancing knowledge and competencies in NCD prevention.

Objective of this review

The systematic literature review aims to evaluate the effectiveness of training interventions in improving the knowledge and competencies of community health workers and community health officers for the prevention and control of non-communicable diseases. It also aims to examine the potential role of innovative educational approaches, particularly mind mapping and mobile-based learning, in strengthening training programs in primary healthcare settings.

## Review

Methodology

Study Design

The present study was conducted as a systematic review following the Preferred Reporting Items for Systematic Reviews and Meta-Analyses (PRISMA) guidelines. The review process was designed to ensure a structured, transparent approach to identifying, screening, assessing eligibility, and including relevant studies. The key methodological components recommended by PRISMA were followed throughout the review process.

Search Strategy

A comprehensive literature search was conducted using electronic databases, including PubMed, Scopus, and Google Scholar. The search included studies published between 2013 and 2025 to ensure coverage of recent and relevant evidence in the field, and the final search was updated on 25 February 2026. The search focused on literature related to community-based interventions and educational strategies for non-communicable disease (NCD) prevention. The search strategy involved combinations of keywords such as “non-communicable diseases,” “community health workers,” “community health officers,” “primary healthcare,” “training,” “mind mapping,” “mobile learning,” and “health education,” which were combined using Boolean operators (AND, OR) to refine the results. An example of the search strategy used in PubMed included: (“non-communicable diseases” OR NCDs) AND (“community health workers” OR “community health officers”) AND (training OR education OR “mind mapping” OR “mobile learning”). To improve relevance, the search was restricted to studies directly related to community health workers, NCD prevention, and training or educational interventions, while studies not aligned with these domains were excluded during screening. In addition to database searches, the reference lists of selected articles were manually screened to identify additional relevant studies.

Study Selection

All identified records were imported and screened in a stepwise manner. Duplicate studies were removed initially, followed by screening of titles and abstracts to assess relevance to the study objectives. Full-text articles were then evaluated for eligibility based on predefined criteria. The study selection process was conducted by the authors, and any discrepancies were resolved through discussion and consensus, without formal calculation of inter-reviewer agreement.

Eligibility Criteria

Studies were included if they focused on community health workers, community health officers, or similar frontline health personnel and reported interventions related to NCD prevention, screening, management, or health education. Eligible studies included a range of designs such as randomized controlled trials, quasi-experimental studies, cross-sectional studies, qualitative studies, and mixed-methods research. Studies were required to report outcomes related to knowledge, competencies, screening practices, communication skills, or health promotion activities. Articles were excluded if they did not focus on NCD-related interventions, lacked sufficient methodological or outcome data, were duplicate publications, or were not available in English.

Data Extraction

Data extraction was conducted using a structured format to ensure consistency and accuracy across studies. Extracted information included author details, year of publication, study design, characteristics of the study population, type of intervention, and key findings. Additional information related to educational approaches and reported improvements in knowledge, screening practices, and communication skills was also recorded. The extracted data were summarized in tables to facilitate comparison and identification of patterns across studies.

Risk of Bias Assessment

The methodological quality of the included studies was assessed using validated risk-of-bias tools appropriate to study design. For randomised controlled trials, the Cochrane Risk of Bias 2 (RoB 2) tool was used, while observational and non-randomised studies were appraised using criteria adapted from the Newcastle-Ottawa Scale (NOS) and ROBINS-I framework. Each study was evaluated across key domains, including selection bias, performance bias, detection bias, attrition bias, and reporting bias for experimental studies, and participant selection, comparability, and outcome assessment for observational studies. Based on these domain-level assessments, studies were categorized as having low, moderate, or high risk of bias. The final risk classification was derived from domain-specific judgments rather than overall subjective assessment, ensuring consistency and transparency across different study designs.

Data Synthesis

The findings of the included studies were synthesised using a structured qualitative approach based on thematic framework analysis. Initially, extracted data were systematically coded and organised to identify recurring patterns across studies. These codes were then grouped into broader thematic categories to enable a more transparent and structured synthesis of evidence. The synthesis categorised interventions into three key domains: screening and early detection interventions, training and educational interventions, and behaviour change and health promotion strategies. Screening-related interventions primarily focused on community-based identification of individuals at risk of non-communicable diseases, while training interventions emphasised capacity building of community health workers through various educational approaches, including innovative methods such as mind mapping. Behaviour change interventions addressed lifestyle modification, patient education, and community engagement aimed at reducing NCD risk factors.

The results within each category were compared and synthesised descriptively to identify common trends, strengths, and gaps across studies. Due to substantial heterogeneity in study designs, intervention types, and outcome measures, quantitative synthesis, effect size estimation, and subgroup analysis were not undertaken. The findings are therefore presented as a narrative synthesis supported by thematic organisation, tables, and figures to enhance clarity and interpretability.

Protocol Registration

This review was conducted in accordance with the PRISMA guidelines to ensure a systematic, transparent, and rigorous approach to study identification, selection, and reporting. Although prospective registration in databases such as the International Prospective Register of Systematic Reviews (PROSPERO) is recommended to enhance transparency, it is not a mandatory requirement for all review studies. The present review was not prospectively registered, which is acknowledged as a limitation.

Results

Search Results

**Figure 1:**
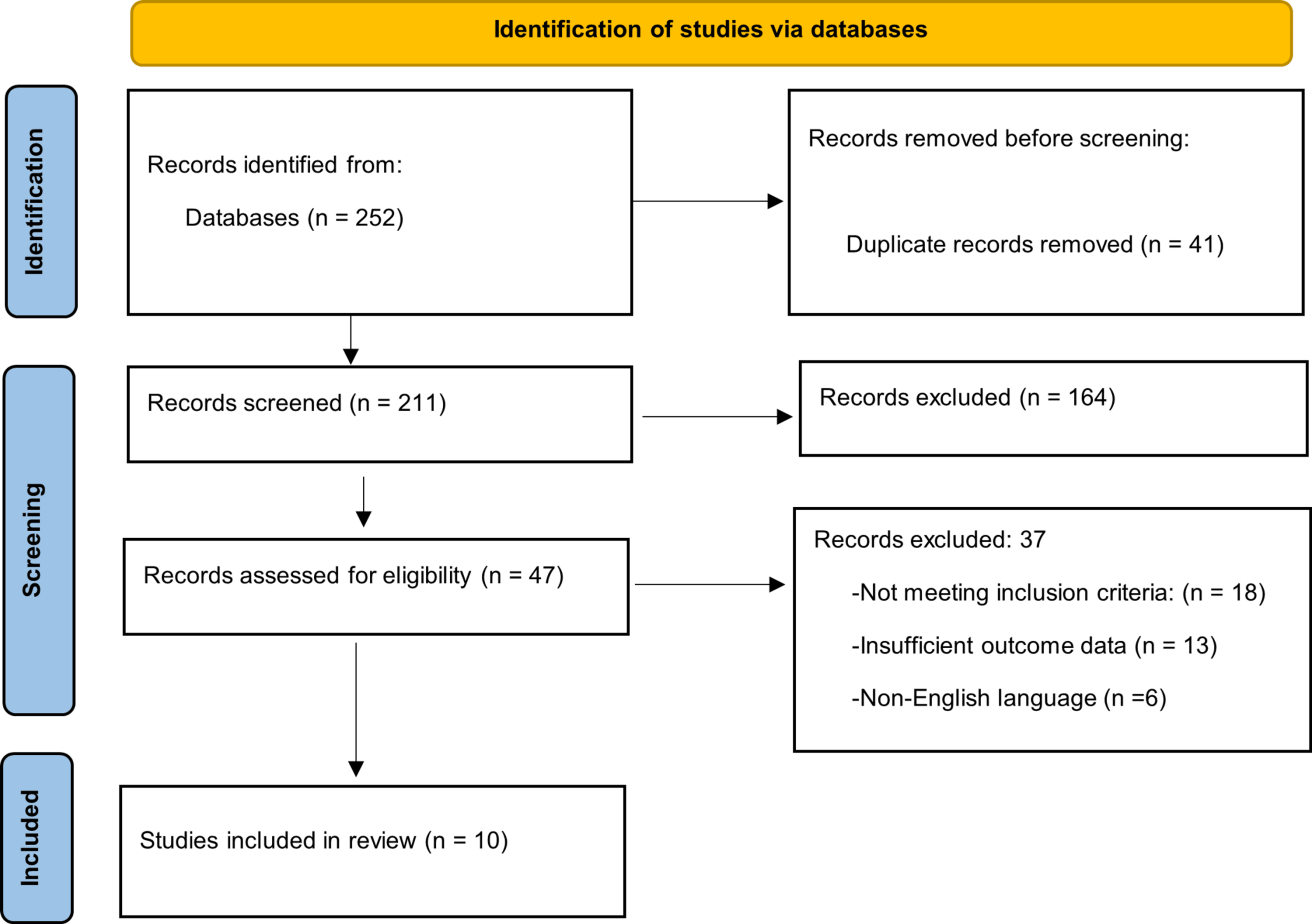
PRISMA flow diagram illustrating the study selection process for inclusion in the systematic review PRISMA: Preferred Reporting Items for Systematic Reviews and Meta-Analyses.

The search process identified studies across multiple electronic databases. A total of 252 records were initially identified through database searching. After removal of duplicate records (n = 41), 211 records remained for title and abstract screening. During this stage, 164 records were excluded as they were not relevant to community health worker interventions or educational strategies for non-communicable disease prevention. The remaining 47 records were assessed for full-text eligibility, of which 37 studies were excluded for specific reasons, including not meeting inclusion criteria (n = 18), insufficient outcome data (n = 13), and non-English language (n = 6). Finally, 10 studies were included in the qualitative synthesis. The detailed study selection process, including identification, screening, eligibility assessment, and final inclusion, is illustrated in the PRISMA flow diagram (Figure 1), ensuring transparency and reproducibility of the selection process.

Study Characteristics

The included studies comprised a range of study designs, including randomised controlled trials, pilot studies, qualitative studies, mixed-method studies, cross-sectional surveys, quasi-experimental studies, and educational intervention studies. To account for variation in methodological rigor, the studies were stratified based on strength of evidence into three categories: high-quality evidence (randomised controlled trials), moderate-quality evidence (quasi-experimental and mixed-method studies), and exploratory evidence (qualitative and cross-sectional studies). Among the 10 included studies, a smaller proportion consisted of randomised controlled trials, while the majority were observational, qualitative, or quasi-experimental in nature, indicating an overall moderate level of evidence.

Most studies focused on the role of community health workers or community volunteers in the prevention and control of non-communicable diseases in settings with limited access to formal healthcare services. The interventions examined included home-based screening, community health promotion, and structured training programs aimed at enhancing the capacity of community health workers to identify and manage risk factors associated with NCDs. The interventions were broadly categorised into three domains: screening and early detection interventions, training and educational interventions, and behaviour change and health promotion strategies. Screening-focused studies primarily evaluated the feasibility and effectiveness of community-based identification of at-risk individuals, while training interventions assessed improvements in knowledge, communication skills, and self-efficacy among healthcare workers. Behaviour change interventions emphasised lifestyle modification, patient education, and community engagement.

Educational approaches investigated across the studies included systematised training, mind mapping, and simulation-based learning, which were associated with improvements in knowledge retention, communication competence, and learner engagement. Stronger and more consistent evidence was observed in high-quality and moderate-quality studies for outcomes such as knowledge improvement and screening effectiveness, whereas findings from exploratory studies provided supportive but less generalisable evidence. Table 1 presents the detailed characteristics of the included studies.

**Table 1:** Characteristics of included studies CHW: community health worker; NCD: non-communicable disease; BFN: barefoot nurse.

Study	Study Design	Participants	Intervention/Focus	Key Outcomes
Basu et al. [21]	Pilot study	Community health workers conducting household screening	Home-based screening for common non-communicable diseases (NCDs)	Demonstrated feasibility of CHW-led household NCD screening and early identification of risk factors in rural populations
Sahu et al. [22]	Randomised controlled trial	Barefoot nurses involved in NCD screening	Barefoot Nurse (BFN) project for NCD screening and livelihood support	Showed the sustainability of the community-based NCD screening model and improved access to screening services
Rawal et al. [23]	Qualitative study	Community health workers	Role of CHWs in NCD prevention and control	CHWs are considered effective for community-level NCD prevention but require training, supervision, and system support.
Mbuthia et al. [24]	Randomised controlled trial	Hypertensive adults receiving CHW support	CHW home-based intervention for hypertension management	Intervention significantly improved blood pressure control compared with usual care
Jabade and Nadaf [25]	Educational experimental study	Nursing students	Mind mapping as a learning technique	Mind mapping improved knowledge retention, information retrieval, and learning engagement among nursing students.
Zaman et al. [26]	Training program evaluation	Community health workers	NCD training program for CHWs	Training significantly improved CHWs’ knowledge and capacity for NCD prevention and health promotion.
Yenit et al. [27]	Cross-sectional study	Health extension workers	Assessment of NCD knowledge, attitudes, behaviour, and self-efficacy	Higher knowledge and positive attitudes are associated with better self-efficacy and healthier behaviours
Pardoel et al. [28]	Mixed-methods training evaluation	Community volunteers	Culturally adapted NCD training program	Training improved volunteers’ knowledge while highlighting challenges in translating knowledge into practice.
Ahmad Fahmy et al. [29]	Mixed-methods program evaluation	Community health volunteers	Capacity-building program for NCD prevention	The program improved volunteers’ knowledge, engagement, and ability to promote healthy behaviours.
Wang et al. [30]	Quasi-experimental study	Postgraduate nursing students (n=74)	Mind mapping combined with standardised patient training	Significant improvement in patient education knowledge, communication competence, self-efficacy, and patient satisfaction

Risk of Bias Assessment

The methodological quality of the included studies was assessed using design-specific risk-of-bias tools. Among the included studies, two randomised controlled trials demonstrated low risk of bias across all assessed domains, indicating high methodological quality. In contrast, the majority of studies (n = 8), including quasi-experimental, qualitative, mixed-method, and cross-sectional designs, were assessed as having moderate risk of bias, primarily due to limitations in participant selection and measurement methods. Selection bias was the most frequently identified limitation, particularly in observational and qualitative studies, while reporting bias was generally low across studies. Measurement bias varied by study design, with moderate concerns observed in studies relying on self-reported outcomes.

To account for differences in study design, the evidence was stratified into three levels: high-quality evidence (randomised controlled trials), moderate-quality evidence (quasi-experimental and educational intervention studies), and exploratory evidence (qualitative and cross-sectional studies). Overall, the evidence base is characterised by moderate methodological quality, with stronger evidence supporting knowledge and screening outcomes from controlled studies, and more variable evidence for behaviour change outcomes from exploratory designs. A summary of the risk of bias assessment across studies is presented in Table 2.

**Table 2:** Risk of bias assessment

Study	Study Design	Selection Bias	Measurement Bias	Reporting Bias	Overall Risk
Basu et al. [21]	Pilot study	Moderate	Low	Low	Moderate
Sahu et al. [22]	Randomised controlled trial	Low	Low	Low	Low
Rawal et al. [23]	Qualitative study	Moderate	Moderate	Low	Moderate
Mbuthia et al. [24]	Randomised controlled trial	Low	Low	Low	Low
Jabade and Nadaf [25]	Educational experiment	Moderate	Low	Low	Moderate
Zaman et al. [26]	Training evaluation	Moderate	Moderate	Low	Moderate
Yenit et al. [27]	Cross-sectional study	Moderate	Moderate	Low	Moderate
Pardoel et al. [28]	Mixed-methods study	Moderate	Moderate	Low	Moderate
Ahmad Fahmy et al. [29]	Mixed-methods evaluation	Moderate	Moderate	Low	Moderate
Wang et al. [30]	Quasi-experimental study	Moderate	Low	Low	Moderate

Community Health Worker-Led Interventions for NCD Prevention and Screening

Several studies highlighted the role of community health workers in improving access to non-communicable disease prevention services through household screening, lifestyle counselling, and early risk identification. Screening and early detection interventions consistently demonstrated improvements in screening coverage and in the identification of at-risk individuals, with moderate strength of evidence supported by both experimental and observational studies. Improvements in knowledge and awareness were also commonly reported, although primarily supported by moderate-quality evidence. However, the magnitude of these improvements was not uniformly reported across studies.

**Figure 2:**
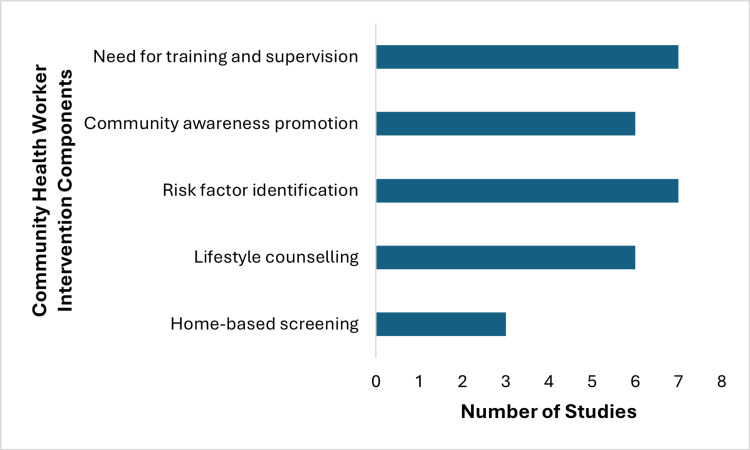
Community health worker intervention components across studies

These interventions also contributed to increased awareness of key risk factors such as hypertension, poor diet, and physical inactivity, particularly in rural and resource-constrained settings. Outcome-wise, evidence for screening uptake and knowledge improvement was more consistent, whereas behaviour change outcomes were less frequently assessed and showed variable and weaker evidence. Overall, variability in study design and outcome reporting limits direct comparison of effectiveness as shown in Figure 2.

Educational and Capacity-Building Interventions for Improving Knowledge and Competence

Educational interventions focusing on capacity building of community health workers and healthcare trainees demonstrated improvements in knowledge, communication skills, and self-efficacy. Outcome-wise, improvements in knowledge and competencies were the most consistently reported findings across studies, supported by moderate strength of evidence from training evaluations and quasi-experimental designs. However, the magnitude of improvement and long-term retention were not consistently quantified.

**Figure 3:**
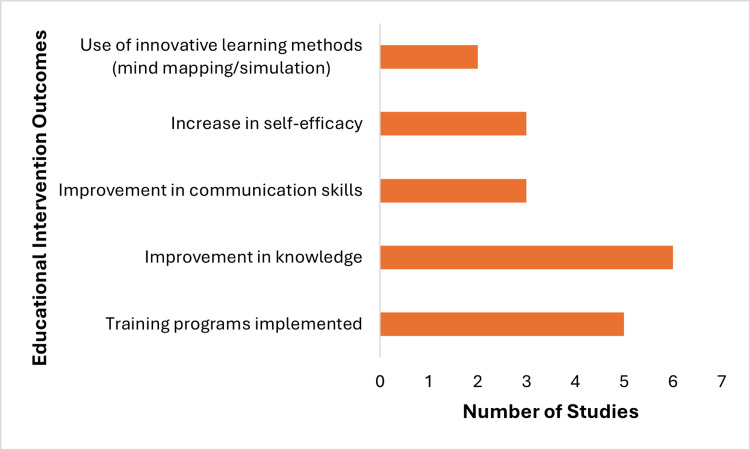
Educational intervention outcomes across studies

Innovative approaches such as mind mapping and simulation-based learning enhanced cognitive organisation, critical thinking, and communication abilities. These methods showed relatively stronger evidence for improving knowledge and communication outcomes compared to behaviour change outcomes, which were less frequently assessed and showed variable results. Overall, while educational interventions are effective in improving knowledge and competencies, evidence for translation into sustained behaviour change or clinical outcomes remains limited, and variability in study design restricts direct comparison of effectiveness. Figure 3 presents the outcomes of these educational interventions.

Discussion

The findings of this review highlight the significant role of community health workers and community health officers in strengthening primary healthcare systems for the prevention and control of non-communicable diseases [28]. These findings are consistent with existing literature demonstrating that community-based health workers contribute effectively to early detection, improved access to care, and enhanced patient follow-up in low-resource settings [29]. Their direct engagement with communities enables early identification of at-risk individuals and timely referral to healthcare facilities. Training interventions tailored to community health workers were associated with improvements in knowledge and practical competencies required for screening, counselling, and disease management. This aligns with prior studies indicating that structured training programs enhance workforce capacity and improve delivery of preventive health services [30], while also strengthening trust and engagement within community health systems [31].

Capacity building remains a critical component in improving the effectiveness of community health workers in NCD prevention and management [32]. Continuous professional development supports sustained delivery of preventive services and strengthens community engagement [33,34]. From a policy perspective, these findings suggest the need for integration of structured and continuous training programs into primary healthcare systems, particularly in low- and middle-income settings. The review also emphasises the importance of innovative educational approaches. Mind mapping was found to enhance cognitive organisation and knowledge retention [35,36], while mobile learning offers flexible and accessible training opportunities, particularly in resource-constrained settings [37]. These findings are supported by existing evidence in health professions education, which highlights the effectiveness of active and technology-enabled learning approaches in improving learning outcomes [38,39]. 

The integration of mobile-based learning with mind mapping represents an emerging approach in healthcare education. While mind mapping supports visual organisation and cognitive processing of complex information, mobile learning enables flexible and continuous access to training resources. Evidence from the included studies and related literature suggests that these approaches independently improve knowledge and engagement; however, their combined application in community health worker training remains limited. This gap highlights an important area for future research, particularly in designing integrated training models that leverage both digital accessibility and visual learning strategies to enhance knowledge retention, decision-making, and practical application in NCD prevention. However, despite growing interest in these methods, there remains limited evidence on their integrated application in training programs for community health officers [40]. This indicates a critical gap in the literature and highlights the need for further research to evaluate combined approaches and their impact on healthcare delivery and patient outcomes.

Limitations and future directions

This systematic review has a few limitations. First, the number of studies specifically addressing the integration of mobile learning and mind mapping in community health worker training was limited, restricting the strength of the conclusions. Second, the included studies were heterogeneous in terms of design, population, and outcome measures, which limited comparability and precluded quantitative synthesis. Third, the overall strength of evidence was moderate, with a predominance of observational and quasi-experimental studies and limited high-quality randomised controlled trials. Fourth, the review included only English-language publications, which may introduce language bias. Additionally, the review relied on published literature, raising the possibility of publication bias and exclusion of relevant grey literature.

Future research should focus on rigorous evaluation of integrated training approaches combining mobile learning and mind mapping, particularly through well-designed randomised controlled trials and implementation studies. Further studies are also needed to assess outcome-specific effectiveness, including knowledge retention, screening uptake, and sustained behaviour change, as well as scalability and feasibility in diverse primary healthcare settings. From a policy and practice perspective, research should explore how digital and visual learning tools can be systematically incorporated into national training frameworks for community health workers to strengthen primary healthcare systems and improve NCD outcomes.

## Conclusions

The findings of this systematic literature review suggest that community health workers and community health officers play an important role in strengthening primary healthcare systems for the prevention and management of non-communicable diseases. Evidence from the included studies indicates improvements in knowledge, screening practices, and health promotion activities following training and capacity-building interventions; however, these findings are primarily supported by moderate-quality evidence and heterogeneous study designs. Empowering community-level health workers may contribute to improved delivery of preventive and promotive services, although the extent of impact varies across settings and interventions. The review also indicates the potential usefulness of innovative educational approaches in healthcare training. Mind mapping appears to support the organisation of knowledge and learning outcomes, while mobile learning technologies offer flexible and accessible training modalities, particularly in resource-constrained settings. Limited and indirect evidence suggests that integrating mobile learning with mind mapping may enhance training effectiveness; however, this combined approach has not been extensively evaluated in community health settings. Overall, these findings highlight the potential of training and innovative educational strategies to strengthen workforce capacity, but further high-quality research is required to establish their effectiveness and scalability in primary healthcare systems.
